# Prevention of Neurite Spine Loss Induced by Dopamine D2 Receptor Overactivation in Striatal Neurons

**DOI:** 10.3389/fnins.2020.00642

**Published:** 2020-06-23

**Authors:** Peng Zheng, Qian Peter Su, Dayong Jin, Yinghua Yu, Xu-Feng Huang

**Affiliations:** ^1^Illawarra Health and Medical Research Institute (IHMRI) and School of Medicine, University of Wollongong, Wollongong, NSW, Australia; ^2^Institute for Biomedical Materials and Devices (IBMD), Faculty of Science, University of Technology Sydney, Sydney, NSW, Australia; ^3^Jiangsu Key Laboratory of Immunity and Metabolism, Xuzhou Medical University, Xuzhou, China

**Keywords:** schizophrenia, cell penetrating-peptide, D2R–DISC1 complex, synaptic spine, GABA, Neuropeptide Y

## Abstract

Psychosis has been considered a disorder of impaired neuronal connectivity. Evidence for excessive formation of dopamine D2 receptor (D2R) – disrupted in schizophrenia 1 (DISC1) complexes has led to a new perspective on molecular mechanisms involved in psychotic symptoms. Here, we investigated how excessive D2R–DISC1 complex formation induced by D2R agonist quinpirole affects neurite growth and dendritic spines in striatal neurons. Fluorescence resonance energy transfer (FRET), stochastic optical reconstruction microscopy (STORM), and cell penetrating-peptide delivery were used to study the cultured striatal neurons from mouse pups. Using these striatal neurons, our study showed that: (1) D2R interacted with DISC1 in dendritic spines, neurites and soma of cultured striatal neurons; (2) D2R and DISC1 complex accumulated in clusters in dendritic spines of striatal neurons and the number of the complex were reduced after application of TAT-D2pep; (3) uncoupling D2R–DISC1 complexes by TAT-D2pep protected neuronal morphology and dendritic spines; and (4) TAT-D2pep prevented neurite and dendritic spine loss, which was associated with restoration of expression levels of synaptophysin and PSD-95. In addition, we found that Neuropeptide Y (NPY) and GSK3β were involved in the protective effects of TAT-D2pep on the neurite spines of striatal spiny projection neurons. Thus, our results may offer a new strategy for precisely treating neurite spine deficits associated with schizophrenia.

## Introduction

Schizophrenia is a severe mental disorder that affects thinking and behavior. Neuroimaging studies have revealed neurobiological deficits in schizophrenia patients and psychosis has been considered as a disorder of impaired neuronal connections (Rae et al., 2017). Striatal dopamine D2 receptor (D2R) hyperactivity is predominantly responsible for psychosis in schizophrenia (Howes and Kapur, 2009). Administration of the D2R-specific agonist quinpirole can induce “hallucinatory-like” behaviors and cognitive decline in monkeys (Arnsten et al., 1995) and dramatically inhibit neurodevelopment of cortical and hippocampal neurons (Reinoso et al., 1996; Jia et al., 2013). Although these studies suggest that striatal D2R hyperactivity accounts for the neuro-pathogenesis of psychosis, the mechanism that underlies increased D2R activity remains to be elucidated.

Antipsychotic drugs antagonizing D2R have been a cornerstone of psychotic pharmacotherapy for more than half a century. D2R is highly expressed in the striatum and striatal D2 receptor blockade is considered the most effective mechanism to alleviate psychotic symptoms in schizophrenia. However, blockade of striatal D2R by antipsychotic drugs is highly likely to cause side effects, including tardive dyskinesia and obesity (Lally and MacCabe, 2015; Huang et al., 2018). We previously reported that both aripiprazole and haloperidol decrease excessive D2R– disrupted in schizophrenia 1 (DISC1) complex formation (Zheng et al., 2018). Further, aripiprazole shows better protection of neurite growth than haloperidol in cortical neurons (Zheng et al., 2018). However, the role of D2R–DISC1 complexes in spine density and neuronal morphology of striatal neurons is as yet unknown.

The molecular composition and organization of synapses are important for synaptic plasticity (Hruska et al., 2018). Stochastic optical reconstruction microscopy (STORM) allows molecules to be assessed at 20 nm resolution, which is sufficient to observe and detect a single molecular interaction (Rust et al., 2006). Recently, STORM imaging revealed that clozapine reverses the changes in D2R assembly in the striatum of DISC1-deficient mice (Onishi et al., 2018). To date, the formation of D2R-DISC1 complexes has not been analyzed in a single synapse. By using STORM and primary striatal neurons, we aimed to reveal the relationship between D2R–DISC1 complexes and dendritic spines from a nanoscale perspective.

Neuropeptide Y (NPY) is one of the most abundant peptides in the central nervous system, with a wide range of physiological functions (Gotzsche and Woldbye, 2016). Most findings regarding the regulation of striatal NPY indicate that there is reciprocal interaction between the D2R and NPY. In fact, D2R activation by administration of quinpirole or amphetamine decreases NPY expression in mice (Kuo, 2003) but the effect of D2R–DISC1 complexes on NPY has not yet been reported.

Cell-penetrating peptide delivery system is highly effective for small peptide delivery in both *in vitro* and *in vivo* studies (Guidotti et al., 2017). It is that the *trans-*activating transcriptional activator (TAT) from human immunodeficiency virus 1 could be efficiently taken up by neurons in culture. This technique is used in the present study to deliver the interfering peptide TAT-D2pep aiming to inhibit D2R and DISC1 complex formation.

In the current study, we show that D2R interacts with DISC1 in dendritic spines and is associated with neurite growth and spine density of striatal neurons. TAT-D2pep reduced excessive D2R – DISC1 complex formation in the spines, soma and neurites and prevented dendritic spine loss of mouse striatal neurons. Furthermore, we observed that NPY and GSK3β were involved in the protective effects of TAT-D2pep in the neurite spines of striatal spiny projection neurons.

## Materials and Methods

### Antibodies and Chemicals

The following reagents were purchased: Quinpirole hydrochloride (Sigma Aldrich, St Louis, MO, United States), anti-MAP2 (1:1,000; Sigma Aldrich), anti-PSD-95 (1:1,000; Abcam, Cambridge, United Kingdom), anti-synaptophysin (1:1,000; Sigma Aldrich), anti-GSK3α/β (1:1,000; Cell Signaling Technology, Danvers, MA, United States), anti-phospho-GSK3β (Ser9) (1:1000; Cell Signaling Technology), anti-β-actin (1:5000; Millipore, Bedford, MA, United States), anti-GAD67 (1:800; Cell Signaling Technology), anti-D2R (1:400, Santa Cruz Biotechnology, CA, United States), anti-NPY (1:400, Santa Cruz Biotechnology), Alexa Fluor 568 Phalloidin (Invitrogen, Waltham, MA, United States), Alexa Fluor 488-conjugated goat anti-mouse IgG secondary antibody (1:400; Invitrogen) and Alexa Fluor 568-conjugated goat anti-rabbit IgG secondary antibody (1:400; Invitrogen), Cy3B-NHS (GE Healthcare, NSW, Australia), and Alexa Fluor 647-NHS (Thermo Fisher, Waltham, MA, United States). The region from K_211_ to T_225_ (KIYIVLRRRRKRVNT) of D2R is known to directly interact with DISC1 (Su et al., 2014; Lipina et al., 2018), which is used for designing TAT-D2peps. TAT-D2pep (YGRKKRRQRRR-KIYIVLRRRRKRVNT) and TAT-D2pep-negative control (NC; YGRKKRRQRRR) were synthesized by GenScript (GenScript, Hong Kong Ltd.), and dissolved in ultrapure water to a stock concentration of 10 mM. The molecular weight and purity of TAT-D2pep was analyzed by high performance liquid chromatography (HPLC) and mass spectrometry (MS) before it was applied in experiments (Supplementary Data, Supplementary Figures S1, S2).

### Striatal Neuron Culture

The experimental procedures were approved by the Animal Ethics Committee, University of Wollongong, Australia (AE17/01), and complied with the Australian Code of Practice for the Care and Use of Animals for Scientific Purposes. Dissociated mouse striatal cultures were prepared as previously described (Mao and Wang, 2003). Briefly, striatal cells of postnatal day 0 of C57Bl/6 mice were gently dissociated with a plastic pipette after digestion with 0.5% trypsin (GIBCO, Los Angeles, CA, United States) at 37°C for 30 min. Cells were cultured in Neurobasal medium (GIBCO) containing B27 supplement (GIBCO) and 20 mM glutamine (Sigma Aldrich). After 24 h of culture, 5-fluoro-2′-deoxyuridine (Sigma) was added to a final concentration of 10 μM to repress the growth of glial cells. Cultures were maintained at 37°C in a humidified 5% CO_2_ incubator. Half volume of the culture medium was changed twice a week. After 14 days in culture, cells were treated with quinpirole for 24 h to induce neurite lesion. The final concentration of quinpirole used in this study was optimized based on its effect on the neurite length of striatal neurons and the data was acquired by a Lionheart FX Automated Microscope (BioTek Instruments, Winooski, VT, United States). Addition of 10 μM TAT-D2pep and TAT-D2pep-NC to cultures was performed 30 min prior to quinpirole addition. We did not observe toxic effect as reported previously (Lee et al., 2016).

### HEK-293 Cell Culture and Transfection

As transcriptomics analysis has shown that D2R and DISC1 are barely expressed in HEK-293 cells (Thul et al., 2017), HEK-293 cells were transfected with D2R and DISC1 plasmids to study the effect of D2R on DISC1 and their downstream signaling cascades. HEK-293 cells were grown in Dulbecco’s modified Eagle’s medium (DMEM; Life Technologies, Carlsbad, CA, United States) containing 10% fetal bovine serum and 1% penicillin-streptomycin (Thermo Fisher) at 37°C in 5% CO_2_. On the day before transfection, the culture medium was changed to DMEM containing 10% fetal bovine serum without penicillin-streptomycin. The D2R-EGFP and DISC1-mCherry coding sequences were synthesized by GenScript (GenScript, Hong Kong Ltd.) and sub-cloned into the pcDNA3 vector. Transfection was performed using Lipofectamine 2000 (Life Technologies) according to the manufacturer’s instructions and after 24 h cells were treated with different drugs, as indicated below.

### Immunofluorescence

Cells for immunofluorescence were plated on 13 mm coverslips coated with 0.1 μg/ml poly-d-lysine (Sigma Aldrich) at a final concentration 1.0 × 10^5^ cells/well. Following treatment, cells were fixed in 4% freshly made formaldehyde, permeabilized with 0.3% Triton X-100/phosphate-buffered saline (PBS), and blocked with 5% normal donkey serum (Sigma Aldrich) in PBS. Cells were firstly immunostained with primary antibodies overnight and then with secondary antibodies for 2 h. Cells were viewed using a 63× oil immersion objective on a TCS SP8 confocal microscope (Leica, Mannheim, Germany). The neurite length was measured by manually tracing the neurites using Simple Neurite Tracer in ImageJ software (Rasband, W.S., ImageJ, U.S. National Institutes of Health, Bethesda, MD, United States^1^).

### Fluorescence Resonance Energy Transfer (FRET)

Confocal fluorescence resonance energy transfer (FRET) analysis was performed as described previously (Zheng et al., 2018). In the striatal neurons, anti-D2R-Alexa Fluor 488 was used as a donor dipole, while anti-DISC1-Alexa Fluor 568 was used as an acceptor dipole. In HEK-293 cells, D2R-GFP was used as a donor dipole, while DISC1-mCherry was used as an acceptor dipole. The donor was excited with an argon laser at 488 nm, while the acceptor was excited with a Diode-pumped solid-state (DPSS) laser at 568 nm. Sensitized emission is one of the most used methods for evaluation of FRET efficiencies. Samples were analyzed using the Leica application wizard for FRET sensitized emission (FRET SE).

### Super-Resolution Imaging of D2R and DISC1 in Dendritic Spines

Primary striatal neurons for STORM imaging were plated on μ-slide 8 well glass bottom plates (Ibidi, Martinsried, Germany). Endogenous D2R and DISC1 were fixed, cells were permeabilized and immunolabeled by primary and secondary antibodies conjugated with Cy3B-NHS and Alexa 647-NHS, respectively, as previously described (Du et al., 2016). An imaging buffer (100 mM Tris/HCl pH 8.0, 20 mM NaCl and 10% glucose, all from Sigma-Aldrich) and an oxygen scavenger system (60 mg/ml glucose oxidase and 6 mg/ml catalase, both from Sigma-Aldrich) were used for STORM imaging (Wang et al., 2015) and 140 mM β-mercaptoethanol was added to promote photo-switching. Two-color STORM imaging was sequentially acquired for up to 50,000 frames under the excitation of 647 nm and 561 nm lasers at a power density of 3∼5 kW/cm^2^ and under the photo-activation of a 405 nm laser (Coherent Inc.) with a power density of 0.5 kW/cm^2^ at the sample. STORM image analysis, nearest neighbor distances calculation, drift correction, image rendering, protein nanocluster identification, quantification and image presentation were performed using Insight3 (a gift from Prof. Bo Huang at UCSF), custom-written Matlab (2012a, MathWorks) codes, SR-Tesseler (IINS, Interdisciplinary Institute for Neuroscience), and Image J (Image Processing and Analysis in Java).

### Western Blot

After treatment, cells were immediately collected in lysis buffer containing NP40 (Sigma Aldrich), protease inhibitor cocktail (Sigma Aldrich), 1 mM phenylmethylsulfonyl fluoride (Sigma Aldrich), and 0.5 mM β-glycerophosphate (Sigma Aldrich). Total protein concentrations were determined by the DC-Assay (BioRad, Hercules, CA, United States), and detected using a SpectraMax Plus384 absorbance microplate reader (Molecular Devices, Sunnyvale, CA, United States). Loading buffer was added to the samples, which were loaded onto 10% sodium dodecyl sulfate polyacrylamide gels (Bio-Rad, Hercules, CA, United States), transferred to nitrocellulose membranes (GE Health, Chicago, IL, United States), and incubated with antibodies overnight at 4°C. The blots were then washed and incubated with secondary antibodies for 2 h at room temperature. For visualization, immunoreactivity was detected using enhanced chemiluminescence detection reagents. The blots were scanned with an Amersham Imager 600 RGB (GE Health, Chicago, IL, United States) and densitometry analysis was performed with ImageQuant TL 8.1 Software (GE Health, Chicago, IL, United States).

### Statistical Analysis

GraphPad Prism 7 (GraphPad Software, Inc., La Jolla, CA, United States) was used to calculate the significance between groups. For significance analyses of four groups, *p*-values were determined by one-way analysis of variance (ANOVA) followed by Tukey’s *post hoc* corrections. Data were expressed as mean ± SEM and *p* < 0.05 was considered statistically different.

## Results

### D2R Interacts With DISC1 in Dendritic Spines

**FIGURE 1:**
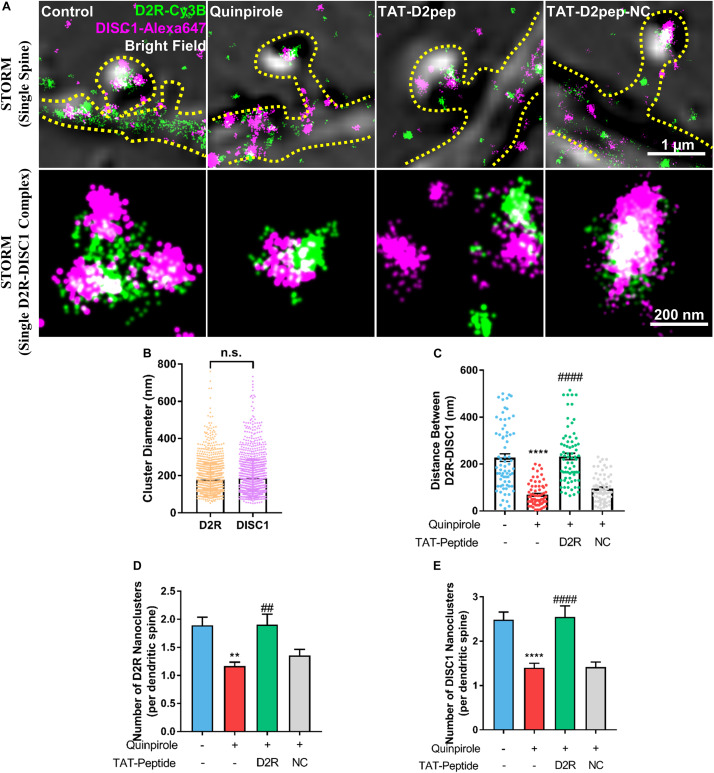
Interaction of D2R–DISC1 in single dendritic spines by STORM analysis. **(A)** Two-color STORM combined with bright field images show the interaction between D2R (green) and DISC1 (red) in single spines. Upper panels show representative images of D2R–DISC1 complex formation in single dendritic spines. Scale bar = 1 μm. Lower panels show magnified representative images of single D2R–DISC1 complexes. Scale bar = 200 nm. **(B)** Diameter of D2R and DISC1 nanoclusters. **(C)** Quantification of the distance between D2R and DISC1 nanoclusters in dendritic spines by the nearest neighbor algorithm. **(D,E)** Number of D2R and DISC1 nanoclusters in single dendritic spines. **P < 0.01 versus control; ****P < 0.0001 versus control; ^##^*P* < 0.01 versus quinpirole; ^####^*P* < 0.001 versus quinpirole by one-way analysis of variance (ANOVA) with *post hoc* Tukey test.

As dendritic spines contain interacting nanomodules, we hypothesized that D2R may interact with DISC1 in dendritic spines. To test this possibility, we firstly immuno-stained D2R and DISC1 with their respective antibodies and then assessed them in dendritic spines by use of STORM. STORM images revealed that D2R and DISC1 molecules exist in dendritic spines and assemble to form nanoclusters with an approximate diameter of 178.6 ± 87.5 nm and 186.1 ± 99.6 nm, respectively (Figures 1A,B). To examine whether D2R–DISC1 complexes were disrupted by TAT-D2pep in spines, we incubated striatal neurons with TAT-D2pep and then analyzed the distance between complexes by applying the nearest neighbor distance algorithm. The nearest neighbor distance between D2R and DISC1 nanoclusters in single dendritic spines was significantly reduced after striatal neurons were treated with quinpirole (control: 227.5 ± 137.5 nm; quinpirole: 69.44 ± 49.06 nm), suggesting the majority of D2R was coupled with DISC1 after treatment. Pre-incubation with TAT-D2pep but not the control TAT-D2pep-NC, significantly enhanced the distance between D2R and DISC1 nanoclusters in the presence of quinpirole (TAT-D2pep: 232.3 ± 118 nm; TAT-D2pep-NC: 95.35 ± 56.95 nm), suggesting that their interaction in dendritic spines was blocked by the interfering peptide (Figure 1C). Notably, when excessive D2R–DISC1 complexes were formed, the number of D2R and DISC1 nanoclusters per dendritic spine were significantly decreased compared with control. The numbers of both D2R and DISC1 nanoclusters were restored after D2R–DISC1 complexes were disrupted by TAT-D2pep, suggesting that D2R–DISC1 complexes affect the D2R and DISC1 densities in dendritic spines (Figures 1D,E). Together, these nanoscale findings demonstrated that TAT-D2pep inhibits excessive D2R–DISC1 complex formation caused by D2R over-activation in dendritic spines.

### TAT-D2pep Inhibits Neurite Loss Caused by Excessive D2R–DISC1 Complexes

**FIGURE 2:**
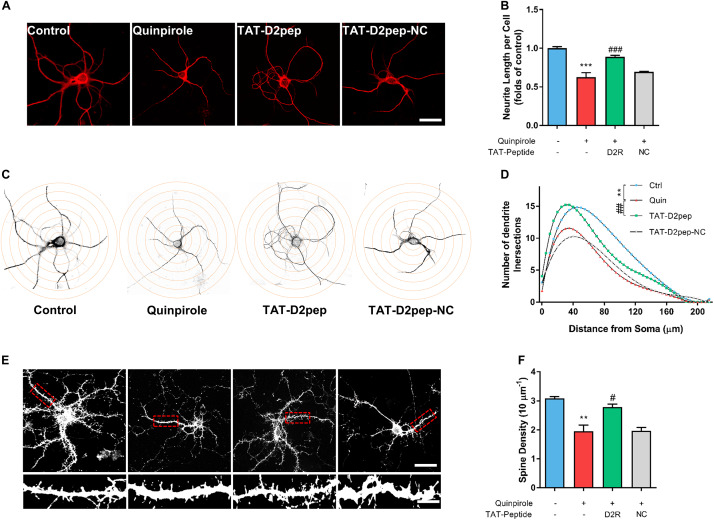
TAT-D2pep protects synaptic spine density and dendritic complexity against D2R hyperactivity in cultured striatal neurons. **(A)** Representative images of striatal neurons immunostained with MAP-2. Scale bar = 50 μm. **(B)** Effect of TAT-D2pep on neurite length. TAT-D2pep inhibited the decrease in neurite length of striatal neurons. **(C,D)** *Sholl* analysis of cultured striatal neurons treated with quinpirole and TAT-D2pep. Representative images of striatal neurons with superimposed circles at given distances from the center of the soma. For *Sholl* analysis, the total number of neurite crossings was counted at each circle with the radius increasing in steps of 5 μm. **P < 0.01 versus control; ^##^*P* < 0.01 versus quinpirole. **(E)** Representative images with high magnification of dendrites immunostained with phalloidin-actin Scale bar = 50 μm (top panels) and 10 μm (bottom panels). **(F)** quantification of spine density. **P < 0.01 and ***P < 0.001 versus control, ^#^*P* < 0.05 and ^###^*P* < 0.001 versus quinpirole by one-way analysis of variance (ANOVA) with *post hoc* Tukey test.

We recently reported that aripiprazole, an atypical antipsychotic drug, protects the neurite length of cortical neurons by blocking D2R signaling and uncoupling D2R–DISC1 complexes (Zheng et al., 2018). To determine whether decreasing abnormal D2R–DISC1 complexes protects neurites, we pre-treated striatal neurons with TAT-D2pep or TAT-D2pep-NC prior to addition of quinpirole, followed by immunostaining for MAP-2 to visualize the effect of D2R–DISC1 complexes in neurites. The concentration of quinpirole required to induce neurite impairment was screened in primary striatal neurons prior to this experiment (Supplementary Figure S3). D2R over-activation induced by quinpirole significantly decreased neurite length compared with control. TAT-D2pep pre-treatment, but not TAT-D2pep-NC, prevented impairment of neurites in striatal neurons (Figures 2A,B). To evaluate the effects of TAT-D2pep on morphology of dendrites in whole neurons, we performed *Sholl* analysis in striatal neurons treated with or without TAT-D2pep. Intersections of dendrites arising from the soma were determined within concentric shells of increasing diameter (5 μm) starting from the cell body (Figure 2C). There was a significant increase of dendritic intersections in the TAT-D2pep treatment group compared with quinpirole group (Figure 2D). To investigate whether TAT-D2pep inhibits dendritic spine loss, we labeled cultured striatal neurons with phalloidin-actin, a marker for spine analysis. Quinpirole significantly reduced synaptic spine density of striatal neurons, suggesting that excessive D2R–DISC1 complexes may cause dendritic spine loss (Figures 2E,F). TAT-D2pep blocked the negative effect of quinpirole on dendritic spine density, suggesting that uncoupling the abnormal formation of D2R–DISC1 complexes may protect spine density of striatal neurons (Figures 2E,F). Taken together, our results suggest that preventing D2R–DISC1 complex formation can block D2R over activation-induced impairment of dendritic morphology and synaptic spine density in striatal neurons.

### TAT-D2pep Uncouples Excessive D2R–DISC1 Complexes Caused by D2R Hyperactivity

**FIGURE 3:**
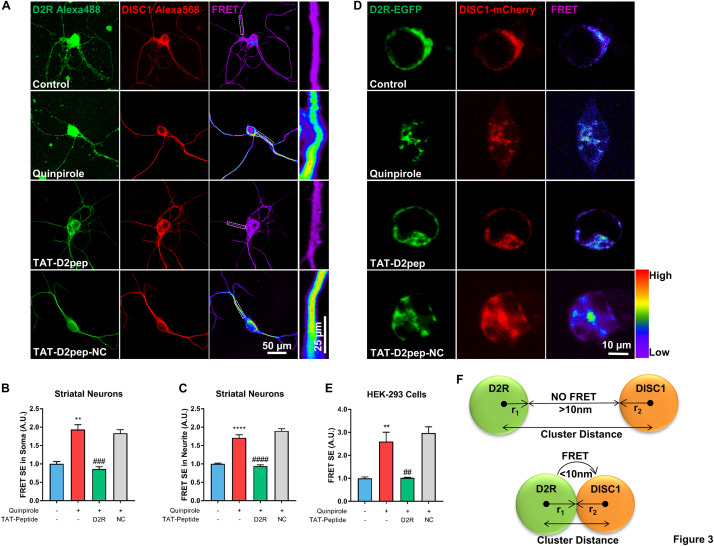
TAT-D2pep specifically blocks excessive D2R–DISC1 complex formation induced by D2R hyperactivity. **(A–C)** FRET analysis showed that TAT-D2pep decreased D2R–DISC1 complex formation induced by quinpirole in soma and neurites of striatal neurons. In **(A)**, the boxes in the FRET images are enlarged in the panels to the right. **(D,E)** FRET images of HEK-293 cells transfected with D2R-EGFP and DISC1-mCherry. D2R hyperactivity-induced D2R–DISC1 complex formation was abolished by TAT-D2pep but not control peptide. Scale bar = 10 μm. **(F)** Schematic relationship between FRET and nanocluster distance: r_1_: radius of D2R nanocluster; r_2_: radius of DISC1 nanocluster. If the D2R–DISC1 nanocluster distance is below the sum of r_1_ + r_2_ + 10 nm, FRET will occur; If the D2R–DISC1 nanocluster distance is above the sum of r_1_ + r_2_ + 10 nm, FRET will not be detected. Data are shown as means ± SEM and normalized; **P < 0.01 and ****P < 0.0001 versus control, ^##^*P* < 0.01, ^###^*P* < 0.001 and ^####^*P* < 0.0001 versus quinpirole by one-way analysis of variance (ANOVA) with *post hoc* Tukey test; *n* > 30 cells per group.

We previously demonstrated that blockade of D2R by its antagonists prevents excessive D2R–DISC1 complex formation induced by quinpirole in mouse cortical neurons (Zheng et al., 2018). As both D2R and DISC1 are expressed in neurites and TAT-D2pep has neuroprotective effects, we next assessed whether TAT-D2pep decreases interaction of D2R and DISC1 in neurites and soma of primary striatal neurons by using FRET. Neurons preincubated with TAT-D2pep, but not TAT-D2pep-NC, showed decreased FRET efficiency in soma and neurites, suggesting that D2R–DISC1 complexes were uncoupled by the interfering peptide (Figures 3A–C). To further determine whether synthesized TAT-D2pep interferes with complex formation, we initially examined D2R–DISC1 interaction using a FRET bioassay in HEK-293 cells expressing D2R-EGFP and DISC1-mCherry. In this experiment, the existence of FRET between D2R-EGFP and DISC1-mCherry was used as an index of the interaction of the two fusion proteins. After addition of quinpirole the FRET efficiency increased more than three-fold compared with HEK-293 cells without quinpirole treatment. The FRET increase was markedly diminished by TAT-D2pep. However, TAT-D2pep-NC, a peptide without the binding domain of D2R and DISC1, failed to decrease FRET efficiency, suggesting the specificity of the designed peptide in uncoupling the D2R–DISC1 interaction (Figures 3D,E). The relationship between the FRET signal and the distance between D2R and DISC1 in dendritic spines, combined with the above super-resolution analysis, is illustrated in Figure 3F. When D2R was over-activated, the nearest neighbor distance between D2R and DISC1 became less than 70 nm, forming a complex in a single dendritic spine and resulting in a FRET signal. These results indicated that TAT-D2pep can specifically inhibit excessive D2R–DISC1 complex formation caused by D2R hyperactivity.

### TAT-D2pep Protects Neurites and Increases Synaptic Protein Expression

**FIGURE 4:**
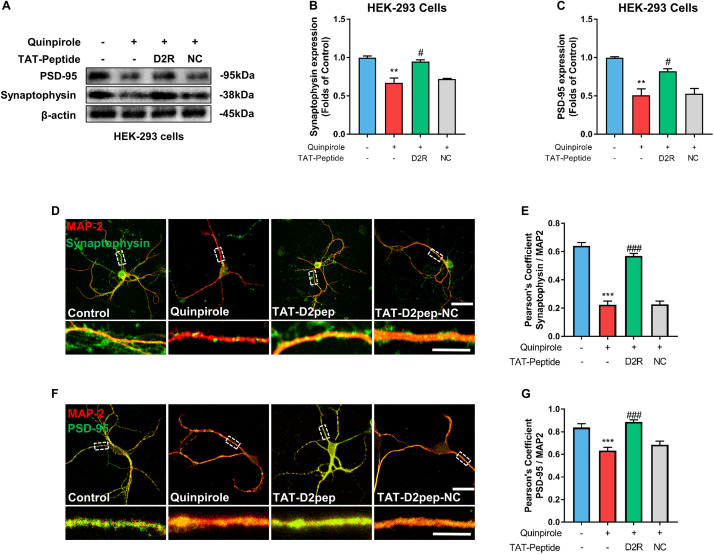
TAT-D2pep blocks D2R hyperactivity-induced down-regulation of synaptophysin and PSD-95. **(A–C)** Western blot analysis showed that TAT-D2pep but not TAT-D2pep-NC increased expression of synaptophysin and PSD-95 in HEK-293 cells co-expressing D2R-EGFP and DISC1 mCherry; *n* > 3 per group. **(D,E)** Representative images of striatal neurons co-immunostained with MAP-2 and synaptophysin. Pearson’s correlation coefficients values for MAP-2 and synaptophysin colocalization assays. **(F,G)** Representative images of striatal neurons co-immunostained with MAP-2 and PSD-95. Pearson’s correlation coefficients values for MAP-2 and PSD-95 colocalization assays. Scale bars = 50 μm and 15 μm (enlarged), **P < 0.01 and ***P < 0.001 versus control, ^#^*P* < 0.05 and ^###^*P* < 0.001 versus quinpirole by one-way analysis of variance (ANOVA) with *post hoc* Tukey test.

As synaptic proteins like synaptophysin and PSD-95 elicit changes in dendritic morphology (Zhang et al., 2016), we hypothesized that TAT-D2pep protects neurites by regulating synaptic proteins. To determine this possibility, we initially performed immunoblot analysis of synaptophysin and PSD-95 in HEK-293 cells co-expressing D2R-EGFP and DISC1-mCherry in the presence of TAT-D2pep or TAT-D2pep-NC. Quinpirole treatment significantly downregulated synaptophysin and PSD-95 expression, suggesting that excessive D2R–DISC1 interaction could directly affect synaptic proteins. Blocking by TAT-D2pep, but not the TAT-D2pep-NC, inhibited the ability of D2R–DISC1 complexes to decrease synaptophysin and PSD-95 expression in HEK-293 cells (Figures 4A–C). We next examined whether the TAT-D2pep peptide could also alter synaptic proteins in primary striatal neurons co-immunostained with MAP-2 and synaptophysin or PSD-95. The immunocytochemical analysis showed that both synaptophysin (presynaptic marker) and PSD-95 (post-synaptic marker) were obviously decreased, which was accompanied by neurite shortening in striatal neurons treated with 10 μM quinpirole. When striatal neurons were pre-treated with TAT-D2pep followed by quinpirole, the synaptophysin and PSD-95 immunofluorescence signals remained at control levels and were accompanied by normal neurites (Figures 4D–G). Therefore, these results indicated that synaptophysin and PSD-95 were associated with the protective effects of TAT-D2pep on neurite and synaptic spine density.

### NPY Positive Interneurons as a Novel Target for Neuroprotection

**FIGURE 5:**
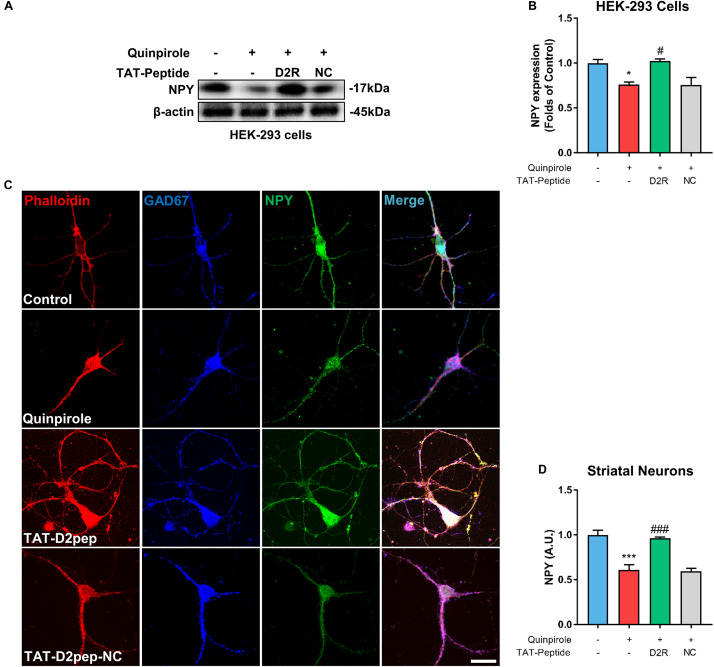
TAT-D2pep protects neurites through upregulating NPY in striatal GABAergic neurons. **(A,B)** Western blot analysis showed that TAT-D2pep but not TAT-D2pep-NC inhibited downregulation of NPY in HEK-293 cells expressing D2R-EGFP and DISC1 mCherry; *n* > 3 per group. **(C,D)** Triple-immunostaining for phalloidin-actin, NPY and GAD67 in primary striatal neurons. TAT-D2pep blocked quinpirole-induced downregulation of NPY in striatal GABAergic neurons, accompanied by normal neurite growth. *P < 0.05 and ***P < 0.001 versus control, ^#^*P* < 0.05 and ^###^*P* < 0.001 versus quinpirole by one-way analysis of variance (ANOVA) with *post hoc* Tukey test.

Neuropeptide Y is mainly expressed in GABAergic neurons and plays an important role in neurite protection (Hokfelt et al., 2008). Thus, we first assessed whether the D2R–DISC1 complex formation induced by D2R over-activation affects NPY expression by performing immunoblot analysis of NPY in HEK-293 cells. When cells were treated with the D2R agonist quinpirole, NPY levels were markedly decreased in lysates of HEK-293 cells co-expressing D2R-EGFP and DISC1-mCherry compared with untreated cells. The decreased NPY levels were inhibited by TAT-D2pep (Figures 5A,B). This result indicates that excessive D2R–DISC1 complex formation caused by D2R over-activation may down-regulate NPY expression. Next, we asked whether NPY is associated with the effects of D2R–DISC1 complexes on neurite growth of striatal spiny projection neurons. Striatal neurons were triple-immunostained for phalloidin-actin, GAD-67 (GABAergic marker) and NPY after quinpirole treatment. Quinpirole significantly decreased NPY expression in striatal GABAergic interneurons, which was accompanied by neurite impairment. The reduction in NPY expression in striatal spiny neurons was significantly inhibited by pre-incubating with TAT-D2pep, but not with the control TAT-D2pep-NC peptide (Figures 5C,D). Thus, theses data demonstrated that the decreased excessive formation of D2R–DISC1 complex upregulated NPY in striatal spiny neurons.

### TAT-D2pep Protects Neurites Through Regulating pGSK3β

**FIGURE 6:**
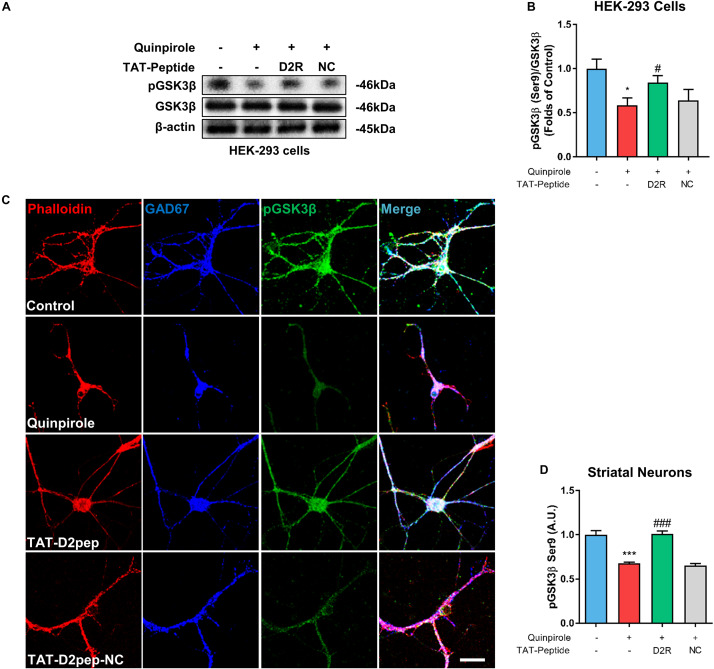
TAT-D2pep protects neurites through increasing phosphorylation of GSK3β in striatal GABAergic neurons. **(A,B)** Western blot analysis showed that TAT-D2pep increased downregulation of pGSK3β in HEK-293 cells expressing D2R-EGFP and DISC1 mCherry; *n* > 3 per group. **(C,D)** Triple-immunostaining for phalloidin-actin, pGSK3β and GAD67 in primary striatal neurons. TAT-D2pep blocked quinpirole-induced downregulation of pGSK3β in striatal GABAergic neurons, accompanied by normal neurite growth. *P < 0.05 and ***P < 0.001 versus control, ^#^*P* < 0.05 and ^###^*P* < 0.001 versus quinpirole by one-way analysis of variance (ANOVA) with *post hoc* Tukey test.

As an important downstream signaling molecule of D2R, GSK-3β is critically involved in neuronal morphological development, including neurite outgrowth (Kim and Snider, 2011). To determine whether GSK-3β phosphorylation is regulated by D2R–DISC1 complexes, we first performed immunoblot analysis in HEK-293 cells. As shown in Figures 6A,B, GSK3β Ser-9 phosphorylation was significantly reduced in quinpirole-treated cells. The ability of quinpirole to decrease GSK3β Ser-9 phosphorylation was significantly inhibited by TAT-D2pep. Next, we investigated whether GSK-3β was involved in mediating the protective effects of TAT-D2pep on neurites. To address this hypothesis, striatal neurons were triple-immunostained with phalloidin-actin, GAD-67 (GABAergic neurons) and GSK-3β after quinpirole treatment. Pre-incubating with TAT-D2pep prevented the inhibitory effect of quinpirole on GSK3β phosphorylation and neurite growth in striatal spiny projection neurons, suggesting that specifically disrupting D2R–DISC1 complex formation could upregulate GSK3β phosphorylation at Ser-9 (Figures 6C,D). Taken together, these data indicate that GSK-3β Ser-9 phosphorylation is associated with the effects of D2R–DISC1 complexes on neurite growth.

## Discussion

The present study showed that excessive formation of D2R and DISC1 complexes alters intracellular signaling pathways and dendritic spine morphology of striatal neurons. We found that D2R formed a protein complex with DISC1 not only in soma and neurites but also in dendritic spines and caused neurite impairment following D2R over-stimulation. We also showed that TAT-D2pep prevented neurite spine impairment caused by D2R over-activation and was associated with increased NPY and pGSK3β in striatal GABAergic neurons.

In the current study, we firstly identified that D2R interacts with DISC1 in dendritic spines. Hruska et al. recently proposed an important theory that a single synaptic spine is composed of nanomodules, the formation of which scales with the size of the dendritic spines (Hruska et al., 2018). It has been observed that D2R exists in higher order oligomers in mouse brain slices (Onishi et al., 2018). Super resolution images indicated that D2R–DISC1 complexes are potential nanomodules existing in a single spine of striatal neurons. By uncoupling D2R–DISC1 complexes in dendritic spines, we linked the actual nanocluster distance to FRET occurrence, which is the first time FRET has been elucidated at a nanoscale perspective. Our study further revealed that TAT-D2pep may restore dendritic spine density by reducing synaptic D2R–DISC1 complexes. Since dendritic spines have several phenotypes associated with learning and memory (Bourne and Harris, 2007), further research is required to define the effect of D2R–DISC1 complexes on spine morphology. Overall, reducing synaptic D2R–DISC1 interaction by TAT-D2pep protected synaptic spine density.

We also found that TAT-D2pep protects neuronal morphology by precisely uncoupling D2R–DISC1 complexes. Notably, although both haloperidol and aripiprazole are able to diminish D2R–DISC1 complexes, haloperidol shows no effect on neuronal protection (Zheng et al., 2018). It suggests that complete blockade of D2R by antipsychotic drugs reduces D2R and DISC1 interaction, but may not protect neurites, which reflects their insufficiency for cognitive improvement (Huang and Song, 2019). It has been reported that there is increased D2R–DISC1 complex formation in the striatum of post-mortem schizophrenic brains (Su et al., 2014). Here, we demonstrated that TAT-D2pep afforded neuronal protection by decreasing D2R–DISC1 complexes instead of blocking D2R. We found that excessive D2R–DISC complexes caused dendritic spine loss in striatal neurons, indicating that the loss of spine density in striatal neurons may be a phenotype of psychosis caused by D2R hyperactivity. In fact, reduction of spine density of cortical and striatal neurons in a schizophrenia-like animal model has been reported previously (Solis et al., 2009). We further found that TAT-D2pep restored the reduced spine density of D2R over-activated striatal neurons, indicating that the dendritic spines of the striatum could be therapeutic targets of this cell-penetrating peptide. In line with our results, increasing D2R–DISC1 complex formation caused by D2R over-activation has been recently demonstrated to decrease long term potentials in mouse hippocampus, indicating a role in synaptic plasticity (Lipina et al., 2018). To our knowledge, this is the first time that TAT-D2pep has been shown to reverse neurite impairment *in vitro*. Further experiments are required to confirm our findings *in vivo*.

We also found that the excessive formation of D2R–DISC1 complexes caused by D2R over-activation remarkably downregulated synaptophysin and PSD-95 in both HEK-293 cells and primary striatal neurons. By transiently expressing D2R and DISC1 in HEK-293 cells, we provided direct evidence that excessive D2R–DISC1 complexes significantly reduced synaptic protein expression. Synaptophysin and PSD-95 act as important regulators of neurodevelopment and synaptic structure (Park et al., 2013; Zhang et al., 2016). It is possible that PSD-95 may indirectly interact with D2R and affect its downstream signaling pathways. For example, DISC1 binds to PSD-95 to facilitate spine enlargement, and knockdown or loss of DISC1 function decreases spine size (Hayashi-Takagi et al., 2010). When more DISC1 binds to D2R, caused by D2R over-activation, less DISC1 may interact with PSD-95, resulting in the reduction of dendritic spine density. We also showed that TAT-D2pep pre-treatment was able to reverse the decreased synaptophysin expression in striatal neurons. Thus, the improvement of neurite and spine density may be a consequence of the enhancement of PSD-95 and synaptophysin expression.

In the current study, we firstly demonstrated that excessive D2R–DISC1 complexes induced by quinpirole directly decreased NPY expression in HEK-293 cells, which was blocked by TAT-D2pep. In the brain, NPY is mainly expressed in GABAergic interneurons and acts as a modulator of neuroplasticity and synaptic transmission (Gotzsche and Woldbye, 2016). Over-activation of D2R by psychostimulants, such as amphetamine, significantly decreases NPY expression (Kuo, 2003). We recently reported that reduced NPY expression is associated with decreased striatal neurite growth and dendritic spine number (Hu et al., 2018). Here, we found that blocking D2R–DISC1 complexes by TAT-D2pep upregulated NPY immunoreactivity in striatal GABAergic neurons. Our evidence suggests that NPY is involved in the protective effects of TAT-D2pep on neurite and spine density. NPY treatment up-regulates pGSK3β, which is inhibited in the presence of a Y1 receptor antagonist (Wu et al., 2017). We further reported that TAT-D2pep increased GSK3β phosphorylation, likely through NPY up-regulation in striatal GABAergic interneurons. Several pharmacological studies from our group previously demonstrated that atypical antipsychotic drugs, such as olanzapine, significantly increased both NPY mRNA and protein expression levels in a schizophrenia mouse model (Huang et al., 2006; Lian et al., 2014). Thus, TAT-D2pep produces an antipsychotic-like response on GABAergic NPY and GSK3β to protect neuronal morphology.

In the striatum, NPY can affect other neuronal populations via NPY receptors. In particular, abundant NPY-Y1 receptor is reported in the human striatum (Caberlotto et al., 1997). NPY-Y1 receptor is shown to mediate the inhibition of locomotor activity after the administration of NPY into rat brain (Heilig et al., 1988, 1989). Therefore, it is speculated that NPY-Y1 receptor may participate in NPY neurotransmission regulating other striatal neurons and locomotor activity.

Although quinpirole is widely used as D2R agonist for many *in vivo* and *in vitro* studies, it should be noted that the quinpirole at 10 μM concentration could act as a partial agonist on alpha 2A adrenoreceptor (Sanchez-Soto et al., 2018). A reduction of alpha 2A adrenoceptor activity could increase the length and density of dendritic spines (Hu et al., 2008). Also, we cannot exclude the possibility that other receptors may interact with D2R in the striatal neurons, which requires the verification of a series of follow-up pharmacological experiments.

In summary, uncoupling D2R–DISC1 complexes by TAT-D2pep prevents neuronal impairment and upregulates NPY and pGSK3β signaling in striatal neurons. Thus, the D2R–DISC1 complex might be a novel therapeutic target for treating neurite deficits in patients suffering psychosis.

## Data Availability Statement

All datasets generated for this study are included in the article/Supplementary Material.

## Ethics Statement

The animal study was reviewed and approved by the Animal Ethics Committee, University of Wollongong, Australia.

## Author Contributions

PZ and X-FH: conceptualization. PZ, QS, YY, and DJ: data curation. X-FH: funding acquisition, writing – review and editing, and project administration. PZ and QS: methodology. PZ: writing – original draft. All authors contributed to the article and approved the submitted version.

## Conflict of Interest

The authors declare that the research was conducted in the absence of any commercial or financial relationships that could be construed as a potential conflict of interest.

## Footnotes

1 https://imagej.nih.gov/ij/, 1997–2016.

## Supplementary Material

FIGURE S1 Purity of TAT-D2pep by reverse-phase HPLC. The area under the major peak corresponds to the percentage purity. For the TAT-D2pep described by the mass spectrum trace above, TAT-D2pep accounted for 98.835% of the peptide species in the sample.

FIGURE S2 Molecular weight of TAT-D2pep by electrospray ionization mass spectrometry. **(A)** Chemical structural formula of TAT-D2pep. **(B)** TAT-D2pep was ionized into multiple-charged positive states. To calculate the measured molecular weight (MW) of TAT-D2pep, the formula was MW = (m/z)^∗^|z| - z. TAT-D2pep MW = 879.3^∗^|4| - 4 = 3513.20; z represents charge number of the ion.

FIGURE S3 Quinpirole inhibits neurite growth of striatal neurons in a concentration-dependent manner. **(A)** Immunofluorescence of MAP-2 demonstrated that 10 μM quinpirole-induced D2R over-activation reduced neurite length of primary striatal neurons. Scale bar = 25 μm. Photos were taken under a 63× oil objective with zoom of 3.5× (top lane) and 1× (bottom lane). **(B)** Quantification of the average neurite length with different concentrations of quinpirole. ^∗∗∗^*P* < 0.001 versus control; *n* = 12 per group.
