# Supplementary figures and images for: Prevention of Neurite Spine Loss Induced by Dopamine D2 Receptor Overactivation in Striatal Neurons
Source: Front Neurosci. 2020 Jun 23;14:642. doi: 10.3389/fnins.2020.00642 (PMC7324769; doi:10.3389/fnins.2020.00642)

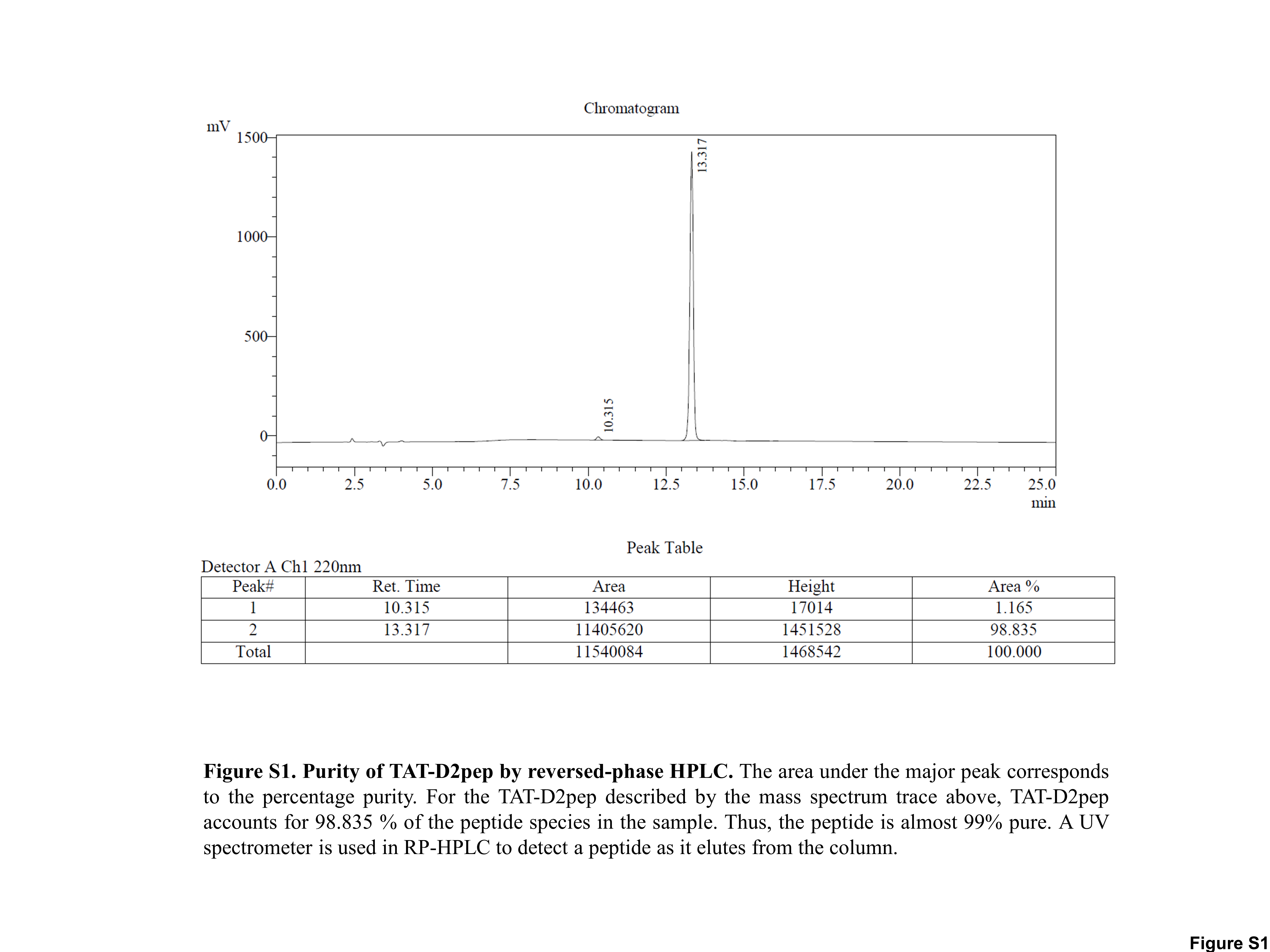

Supplement: FIGURE S1 — Purity of TAT-D2pep by reverse-phase HPLC. The area under the major peak corresponds to the percentage purity. For the TAT-D2pep described by the mass spectrum trace above, TAT-D2pep accounted for 98.835% of the peptide species in the sample. [file Image_1.TIF]

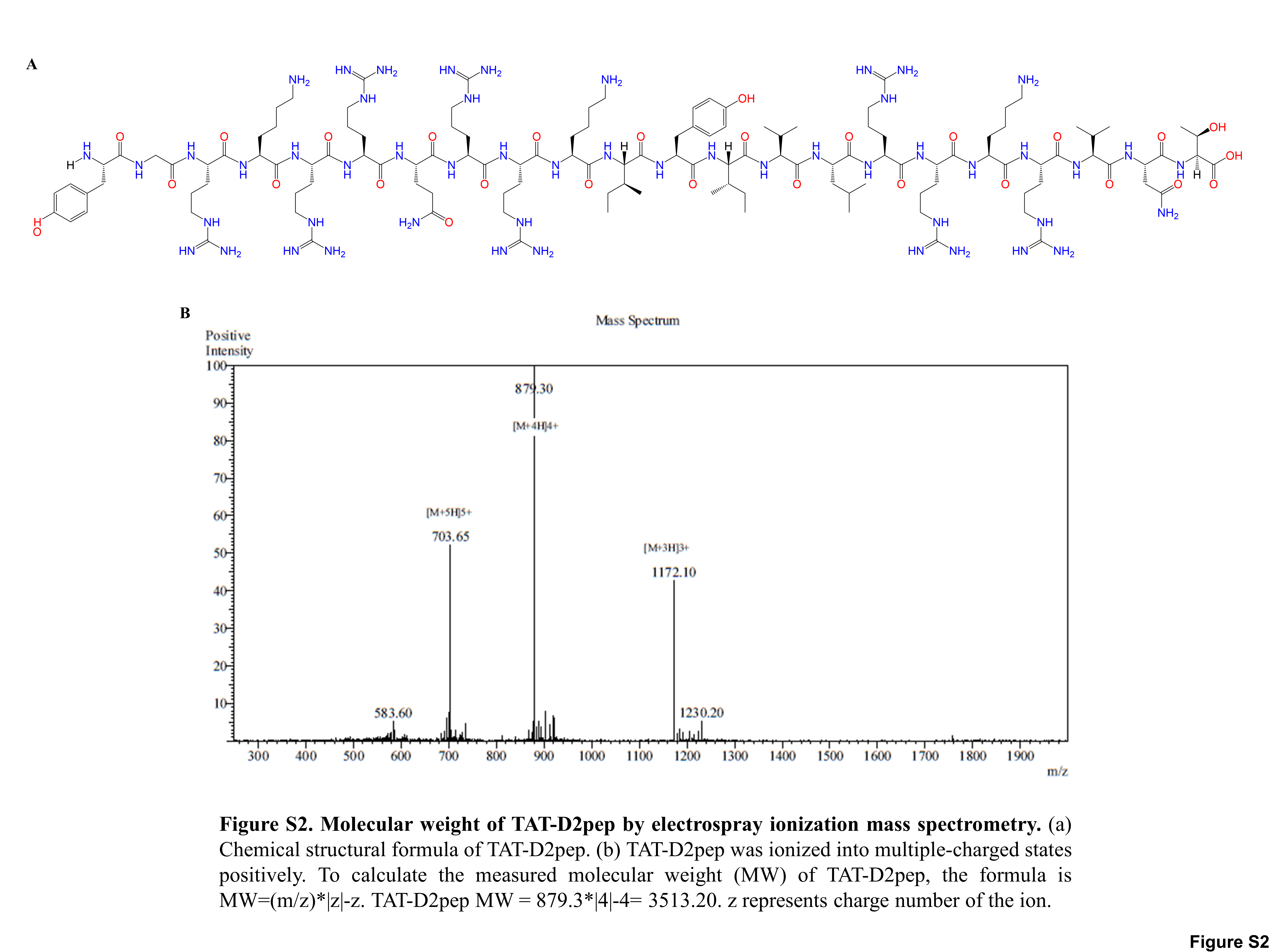

Supplement: FIGURE S2 — Molecular weight of TAT-D2pep by electrospray ionization mass spectrometry. (A) Chemical structural formula of TAT-D2pep. (B) TAT-D2pep was ionized into multiple-charged positive states. To calculate the measured molecular weight (MW) of TAT-D2pep, the formula was MW = (m/z)∗|z| - z. TAT-D2pep MW = 879.3∗|4| - 4 = 3513.20; z represents charge number of the ion. [file Image_2.TIF]

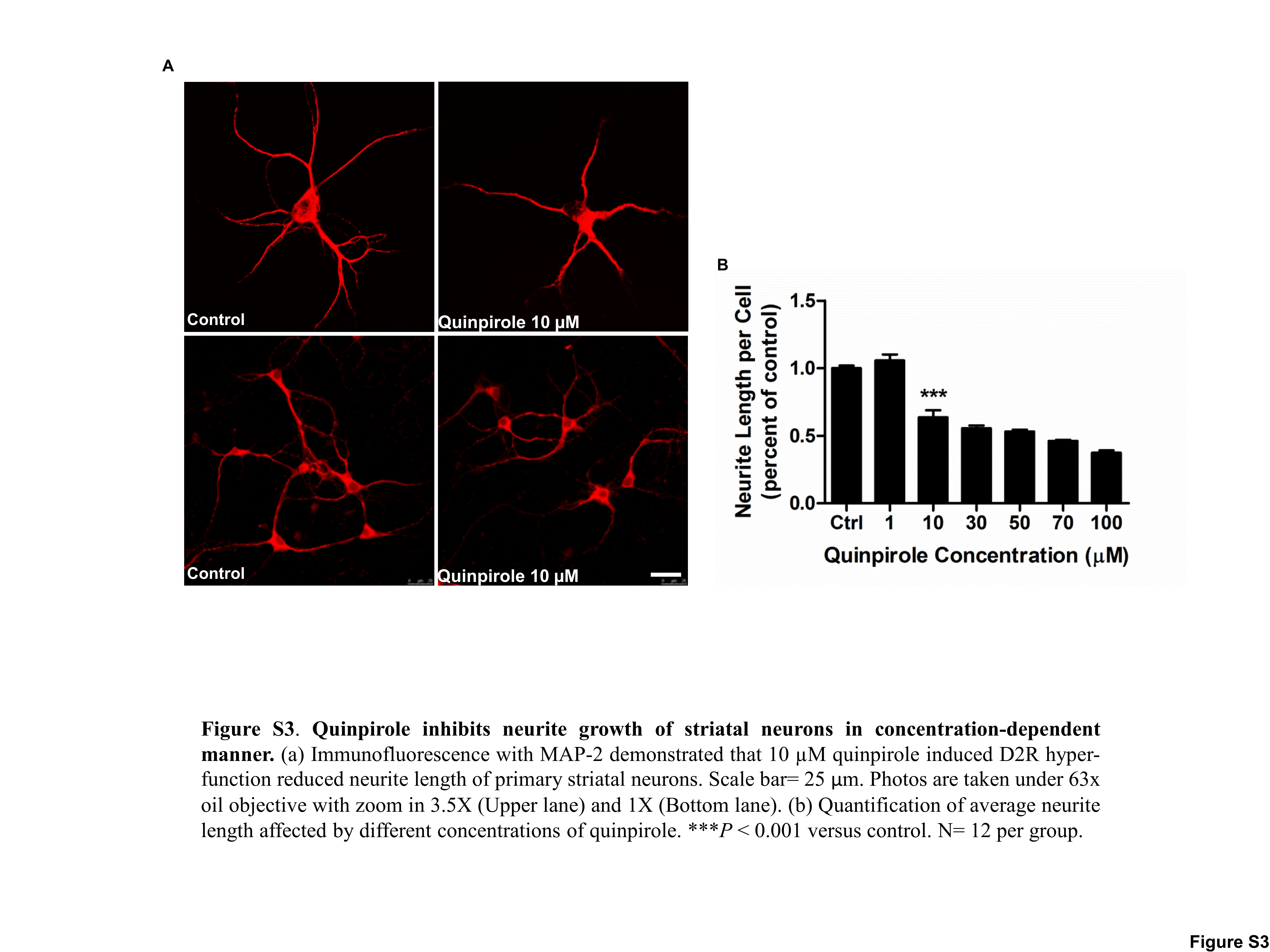

Supplement: FIGURE S3 — Quinpirole inhibits neurite growth of striatal neurons in a concentration-dependent manner. (A) Immunofluorescence of MAP-2 demonstrated that 10 μM quinpirole-induced D2R over-activation reduced neurite length of primary striatal neurons. Scale bar = 25 μm. Photos were taken under a 63× oil objective with zoom of 3.5× (top lane) and 1× (bottom lane). (B) Quantification of the average neurite length with different concentrations of quinpirole. ∗∗∗P < 0.001 versus control; n = 12 per group. [file Image_3.TIF]
